# Implementing PEN‐FAST for penicillin allergy delabeling in a high‐prevalence population

**DOI:** 10.1111/ddg.15862

**Published:** 2025-09-14

**Authors:** Deniz Göcebe, Katharina S. Kommoss, Martin Hartmann, Alexander H. Enk, Knut Schäkel

**Affiliations:** ^1^ Department of Dermatology University Hospital Heidelberg Heidelberg University Heidelberg Germany; ^2^ Division of Infectious Diseases and Tropical Medicine University Hospital Heidelberg Heidelberg University Heidelberg Germany

**Keywords:** Allergy delabeling, drug allergy, PEN‐FAST, penicillin allergy

## Abstract

**Background and objectives:**

Self‐reported penicillin allergies lead to the use of broad‐spectrum antibiotics, increase drug resistance, and constitute an economic burden. The PEN‐FAST score aims to identify low‐risk patients for direct drug provocation tests (DPT) without prior skin testing with a reported negative predictive value (NPV) of over 95%.

**Patients and methods:**

In this single‐center study (Department of Dermatology, University Hospital Heidelberg, Germany), the PEN‐FAST score was evaluated for patients carrying a penicillin allergy label between 2004 and 2024. Skin testing, allergen‐specific IgE, and consecutive DPT were performed.

**Results:**

A total of 189 patients were analyzed. In a retrospective cohort, 106 of 149 patients showed negative skin tests and received DPT, leading to the delabeling of 99 patients (66.4%). PEN‐FAST identified 55 of 149 (36.9%) as low‐risk, of which three low‐risk patients were misclassified. In a prospective PEN‐FAST low‐risk cohort, one of 40 patients showed a mild reaction after DPT. Overall, NPVs of both PEN‐FAST and formal allergy testing were 95.8%.

**Conclusions:**

Our results advocate for direct DPT in patients carrying a penicillin allergy label classified as low‐risk by PEN‐FAST. PEN‐FAST demonstrated high NPV, safety, and feasibility in a cohort with a high prevalence of true allergies.

## INTRODUCTION

Self‐reported penicillin allergy is the most common drug allergy, with a prevalence of approximately 10% in Europe and the United States.^1^ Less than 10% of patients who carry a penicillin allergy label are truly allergic, and cross‐allergies to other beta‐lactam antibiotics mostly constitute a negligible risk.^2^, ^3^ False penicillin allergy labels lead to increased drug resistance, increased probability of adverse events, and prolonged hospitalization, and also constitute an economic burden.^4^, ^5^ A drug provocation test (DPT) with the suspected penicillin is considered the gold standard for removing penicillin allergy labels. However, conventional allergy assessments require skin and allergen‐specific IgE testing before DPT, restricting allergy delabeling to specialized clinics. Given the high prevalence of self‐reported penicillin allergies, there is an urgent need for effective clinical tools to facilitate accurate delabeling of low‐risk allergies by non‐allergist clinicians.

PEN‐FAST is an internationally validated scoring system based on three clinical criteria to identify low‐risk adult patients carrying a penicillin allergy label that are suitable for DPT without prior skin testing.^6^ A PEN‐FAST score < 3 was shown to have a negative predictive value (NPV) of over 95%.^6^ A two‐arm randomized trial recently demonstrated non‐inferiority of PEN‐FAST to skin testing in predicting immediate allergic reactions.^7^ However, the study focused on IgE‐mediated adverse events, and skin testing specifically assessed immediate allergies. Subsequent studies could validate the NPV of PEN‐FAST but were also limited to immediate reactions.^8^, ^9^, ^10^ Conversely, several studies recently questioned the NPV of PEN‐FAST,^11^, ^12^, ^13^ proposing either to lower the threshold^13^ or to modify the score by including additional criteria.^12^ However, the incorrect labeling of PEN‐FAST in these studies was largely attributed to positive skin test results, which are suspected to have low positive predictive values in low‐risk patients.^14^, ^15^

Our study aimed to confirm the initially reported NPV of PEN‐FAST^6^ in a high‐prevalence population for both immediate and delayed penicillin allergies. We compared the performance and safety of PEN‐FAST with routine allergy assessments. Furthermore, we modified the scope of allergy testing to analyze the effect of fewer skin tests on the predictive capacity of PEN‐FAST.

## PATIENTS AND METHODS

### Participants

This monocentric study, combining retrospective and prospective cohorts, was conducted at the Department of Dermatology, University Hospital Heidelberg, Germany.

In a retrospective analysis, outpatients and inpatients admitted to the allergology department with a reported penicillin allergy (immediate, delayed, or unknown/unclassifiable) between January 2004 and April 2024 were analyzed. In a prospective group, inpatients carrying a penicillin allergy label with a PEN‐FAST score < 3 between January 2024 and October 2024 were included.

Key exclusion criteria were age < 18 years, pregnancy, chronic urticaria, mastocytosis, concurrent immunosuppressive therapy with 20 mg of prednisolone per day or steroid equivalent, and incapacity to consent (available in the online supplementary Appendix).

All included patients underwent a full allergology assessment. Patients who were considered allergic based solely on their reported medical history without further testing, patients who refused skin testing or DPT, and patients with a self‐reported penicillin allergy who received DPT with an alternative beta‐lactam antibiotic other than the accused drug were not included in the analysis.

### Procedures

Skin testing included prick and patch tests. No intradermal tests were performed, which can increase sensitivity but may also lead to false‐positive results.^16^ Penicilloyl‐polylysine (PPL) and commercial minor determinants are not available in Germany. Thus, patients in the retrospective group received skin prick tests and patch tests (if a delayed allergy was suspected) with the culprit penicillin following EAACI recommendations for test concentrations.^17^

Total serum IgE and allergen‐specific IgE were measured (ImmunoCAP, Thermo‐Fischer) (supplementary Appendix and supplementary Table S2). Specific/total IgE ratio values were calculated for patients with positive findings in allergen‐specific IgE as previously described^18^ (supplementary Table S3). Tryptase levels were measured to rule out mastocytosis, and SX1 inhalant allergens were analyzed (supplementary Table S2).

In the prospective cohort, patch tests were omitted to eliminate false‐positive results, as all patients falsely delabeled by PEN‐FAST in the retrospective group retained their label due to positive patch tests. The patient history in the prospective group was collected using standardized questionnaires (supplementary Appendix).

Patients with any positive finding in skin tests, allergen‐specific IgE, or DPT were considered allergic. PEN‐FAST scores were determined for each patient according to the original study.^6^ Due to the insignificance to the PEN‐FAST score, we did not differentiate between urticaria and rash (supplementary Appendix). The PEN‐FAST score requires anaphylaxis to show systemic symptoms in addition to cutaneous manifestations,^6^ and patients with urticaria/rash received one point if they reported systemic allergic manifestations.

DPT was performed with the lowest available therapeutical dose of the culprit penicillin with a two‐step challenge (supplementary Appendix). After DPT, patients in the retrospective cohort were either observed overnight on an inpatient basis or for at least 4 hours for outpatients. Patients in the prospective cohort were observed at least overnight on an inpatient basis. The primary endpoint was any positive penicillin allergy result in the above‐mentioned tests. No routine follow‐up was performed. Patients were instructed to contact the hospital if an allergy‐related symptom occurred after being discharged.

### Statistical analysis

Sensitivity, specificity, positive predictive value (PPV), negative predictive value (NPV), receiver operating characteristic (ROC) curves with area under the curve (AUC), and accuracy with exact 95% confidence intervals (CIs) were calculated for the different PEN‐FAST scores. For skin tests and allergen‐specific IgE, NPVs with exact 95% CIs were calculated. The distribution of serological allergy markers was assessed for normality using the D'Agostino‐Pearson normality test. The results demonstrated that the data did not follow a normal distribution (p < 0.05). Consequently, the non‐parametric Kruskal‐Wallis test was performed to compare groups, with a p value of < 0.05 considered statistically significant. Statistical analysis was performed using IBM SPSS statistics version 29 and GraphPad prism 10.

### Ethics

The study was performed according to the *Declaration of Helsinki* and approved by the local ethics committee of the medical faculty of the University of Heidelberg, Germany, under references S‐756/2023 and S‐289/2024. All prospective participants gave written informed consent. Data collected in the retrospective cohort did not require informed consent.

## RESULTS

### Study Population

**FIGURE 1:**
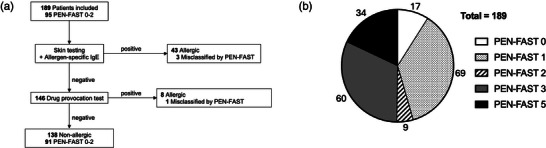
Results of penicillin allergy assessment and PEN‐FAST score. (a) Outcomes of evaluation of penicillin allergy assessment. Patients with any positive finding in skin tests and/or allergen‐specific IgE maintained their allergy label. (b) PEN‐FAST score distribution. The chart indicates the number of patients for each PEN‐FAST score. PEN‐FAST scores 0–2 are defined as low risk (< 5%).

A total of 189 adult patients were included (Figure 1a), with 149 patients in the retrospective and 40 patients in the prospective group (supplementary Figure S1). The study population was predominantly female (74.6%), with a median age of 47 years (interquartile range [IQR] 35–60) (Table 1). The most frequently reported allergy type was a delayed penicillin allergy (Table 1). Most patients reported an allergy either to penicillin VK/G or amoxicillin (47.1% each) (Table 1). The most frequently reported symptoms were urticaria/rash (130 of 189; 68.8%) and angioedema (21 of 189; 11.1%) (Table 1). In total, 95 patients (50.3%) were classified as low‐risk (0–2) by PEN‐FAST, with most patients having a score of one point (Table 1).

**TABLE 1:** Baseline characteristics of included patients.

	Total (n = 189)	Non‐allergic (n = 138)	PEN‐FAST 0–2 (n = 95)
***Sex***			
Male	47 (24.9)	34 (18.0)	21 (11.1)
Female	141 (74.6)	103 (54.5)	74 (39.2)
***Age, median (IQR)***	***47 (35‐60)***	***50 (38‐61)***	***53 (42‐61)***
***Allergy type***			
Immediate allergy	34 (18.0)	16 (8.5)	6 (3.2)
Delayed allergy	82 (43.4)	57 (30.2)	31 (16.4)
Unclear/unknown	73 (38.6)	65 (34.4)	58 (30.7)
***Reported allergy label***			
Penicillin VK/G	89 (47.1)	69 (36.5)	60 (31.7)
Amoxicillin	89 (47.1)	60 (31.7)	31 (16.4)
Ampicillin	7 (3.7)	5 (2.6)	1 (0.5)
Flucloxacillin	2 (1.1)	2 (1.1)	1 (0.5)
Piperacillin	2 (1.1)	2 (1.1)	2 (1.1)
***Reported symptoms***			
Urticaria/rash	130 (68.8)	93 (49.2)	66 (34.9)
Angioedema	21 (11.1)	10 (5.3)	0 (0)
Respiratory symptoms	7 (3.7)	4 (2.1)	0 (0)
Gastrointestinal symptoms	6 (3.2)	6 (3.2)	2 (1.1)
Hypotension	8 (4.2)	3 (1.6)	0 (0)
SCAR	5 (2.6)	2 (1.1)	0 (0)
Unclear/unknown	16 (8.5)	16 (8.5)	16 (8.5)
***Treatment***			
No treatment required	27 (14.3)	26 (13.8)	26 (13.8)
Treatment required for reaction	50 (26.5)	30 (15.9)	5 (2.6)
Local corticosteroids	3 (1.6)	3 (1.6)	1 (0.5)
Oral corticosteroids/antihistamines	18 (9.5)	14 (7.4)	3 (1.6)
IV corticosteroids/antihistamines	27 (14.3)	12 (6.3)	1 (0.5)
Adrenaline treatment	2 (1.1)	1 (0,5)	0 (0)
Unclear/unknown	116 (61.4)	86 (45.5)	64 (33.9)
***PEN‐FAST criteria***			
Five years or less since reaction	95 (50.3)	49 (25.9)	6 (3.2)
Anaphylaxis/angioedema or SCAR	42 (22.2)	22 (11.6)	3 (1.6)
Treatment required for reaction	50 (26.5)	30 (15.9)	5 (2.6)
***PEN‐FAST scores***			
PEN‐FAST 0	17 (9.0)	17 (9.0)	17 (9.0)
PEN‐FAST 1	69 (36.5)	66 (34.9)	69 (36.5)
PEN‐FAST 2	9 (4.8)	8 (4.2)	9 (4.8)
PEN‐FAST 3	60 (31.7)	31 (16.4)	0 (0)
PEN‐FAST 4	0 (0)	0 (0)	0 (0)
PEN‐FAST 5	34 (18.0)	16 (8.5)	0 (0)

Values indicate number of patients (%). PEN‐FAST scores 0–2 are defined as low risk (< 5%).

*Abbr*.: SCAR, severe cutaneous adverse reaction

### Performance of PEN‐FAST

In total, 95 out of 189 patients (50.3%) were assessed as low‐risk by PEN‐FAST (PEN‐FAST score < 3) (Figure 1b) of which four patients were misclassified (Figure 1a and Table 2; detailed characteristics of all misclassified patients can be found in supplementary Table S1). In the retrospective cohort, PEN‐FAST identified 55 of 149 (36.9%) patients at low‐risk (PEN‐FAST score < 3) (supplementary Figure S1), of which three patients had a positive patch test for amoxicillin. PEN‐FAST proved to have good applicability, identifying 52 of 99 (52.5%) non‐allergic patients.

**TABLE 2:** Performances of PEN‐FAST cut‐off scores.

	0 vs. 1–5 (n = 17)	0–1 vs. 2–5 (n = 86)	0–2 vs. 3–5 (n = 95)	0–3 vs. 4–5 (n = 155)	0–4 vs. 5 (n = 155)
Sensitivity (95% CI)	1.0 (0.93–1.0)	0.94 (0.84–0.99)	0.92 (0.81–0.98)	0.35 (0.22–0.50)	0.35 (0.22–0.50)
Specificity (95% CI)	0.12 (0.07–0.19)	0.60 (0.51–0.68)	0.66 (0.57–0.74)	0.88 (0.82–0.93)	0.88 (0.82–0.93)
PPV (95% CI)	0.30 (0.23–0.37)	0.47 (0.37–0.57)	0.50 (0.40–0.60)	0.53 (0.35–0.70)	0.53 (0.35–0.70)
NPV (95% CI)	1.00 (0.80–1.00)	0.97 (0.90–0.99)	0.96 (0.90–0.99)	0.79 (0.71–0.85)	0.79 (0.71–0.85)
Accuracy (95% CI)	0.36 (0.29–0.43)	0.69 (0.62–0.76)	0.73 (0.66–0.79)	0.74 (0.67–0.80)	0.74 (0.67–0.80)
AUC (95% CI)	0.56 (0.50–0.59)	0.77 (0.68–0.84)	0.79 (0.69–0.86)	0.62 (0.52–0.72)	0.62 (0.52–0.72)

*Abbr*.: AUC, area under the curve; CI, confidence interval; NPV, negative predictive value; PPV, positive predictive value

In the prospective cohort that included only patients with a PEN‐FAST score < 3, both skin testing and allergen‐specific IgE were negative in all patients. Following DPT, only one of 40 patients reported a mild cutaneous reaction.

PEN‐FAST showed a high sensitivity of 92.2% (95% CI, 81.1%–97.8%) but a low specificity of 65.9% (95% CI, 57.4%–73.8%) (Table 2). NPV was 95.8% (95% CI, 89.6%–98.8%), while PPV was 50.0% (95% CI, 39.5%–60.5%). A PEN‐FAST cutoff of two points showed moderate discrimination with an AUC of 0.79 (95% CI, 0.69–0.86). Lowering the threshold of PEN‐FAST to 0‐1, as previously suggested,^13^ only resulted in a minor increase in NPV but lowered discrimination, accuracy, and feasibility (Table 2). PEN‐FAST misclassified no patients with a high‐risk history, as all four misclassified patients had reported only urticaria/rash without systemic symptoms. The four misclassified patients were all female, with three subjects reporting an allergy to amoxicillin and one reporting penicillin VK/G (supplementary Table S1).

### Outcomes of serological allergy markers

Total serum IgE levels were significantly higher in allergic patients compared to non‐allergic individuals, with values of 293.1 (± 949.2) and 76.9 (± 106.8), respectively (supplementary Table S2). Allergen‐specific IgE was positive in 9.8% of patients (Table 3). To further evaluate whether increased total‐serum IgE levels affected allergen‐specific IgE, the specific/total IgE ratio was calculated as previously described (supplementary Table 2).^18^ While three patients showed ratios below the suggested threshold of 0.002, two patients were subjected to DPT and one showed an allergic reaction. Serum tryptase and SX1 inhalant allergens showed no significant differences between the PEN‐FAST low‐risk and high‐risk groups as well as between allergic and non‐allergic individuals (supplementary Table S2).

**TABLE 3:** Performances of conventional allergy testing methods.

	Overall (n = 189)	Patch test (n = 114)	Skin prick test (n = 164)	Specific IgE (n = 184)
Positive findings, n (%)	46 (24.3)	25 (21.9)	12 (7.3)	18 (9.8)
Negative findings, n (%)	143 (75.7)	89 (78.1)	152 (92.7)	166 (90.2)
NPV (95% CI)	0.96 (0.91–0.98)	0.85 (0.76–0.92)	0.82 (0.74–0.87)	0.80 (0.73–0.86)

*Abbr*.: NPV, negative predictive value; CI, confidence interval

### Performance of conventional allergy testing

Positive findings in skin testing and allergen‐specific IgE cannot be verified as patients were not subjected to DPT. Hence, the true performance of these tests remains unknown, and only the NPV can be derived. Skin tests and allergen‐specific IgE, which are selective for either immediate or delayed allergies, individually showed poor NPV (Table 3). However, the combined overall NPV of skin tests and allergen‐specific IgE was 95.8% (95% CI, 91.1%–98.4%) (Table 3). Thus, formal allergy testing and PEN‐FAST demonstrated comparable negative predictive capacity, but formal allergy testing facilitated the delabeling of an additional 47 patients assessed at high risk by PEN‐FAST (≥ 3 points). However, formal allergy testing also misclassified five additional patients, including one patient with airway obstruction (supplementary Table S1).

### Allergy testing outcomes

The penicillin allergy label was removed in 138 of 189 subjects (73.0%), while 51 patients were confirmed as allergic (Figure 1a, Table 1). Of the 51 patients who retained their allergy label, 45 individuals were identified by positive skin testing and IgE, respectively (Figure 1a). DPT was performed on 146 patients, with eight patients showing an allergic reaction (Figure 1a), none of which were fatal. Six patients reported only mild cutaneous reactions, one individual reported a bullous reaction, and one patient developed respiratory symptoms following DPT. One patient with a positive patch test and two patients with positive allergen‐specific IgE were subjected to DPT despite positive findings (data not shown). The patient with a positive patch test and one of the subjects with positive allergen‐specific IgE developed an allergic reaction and retained their allergy label, whereas the remaining patient was delabeled despite elevated specific IgE.

## DISCUSSION

PEN‐FAST aims to reduce the number of skin tests needed before DPT and enables non‐allergist clinicians to perform allergy delabeling based on objective clinical criteria. PEN‐FAST was developed and validated as a clinical tool with an NPV exceeding 95%, demonstrating safety comparable to skin testing.^6^, ^7^, ^10^ Our study validates PEN‐FAST as a safe, feasible, and accurate clinical decision rule for assessing both immediate and delayed penicillin allergies. PEN‐FAST showed an NPV of > 95% in a population with a higher prevalence (33.6% in the retrospective cohort and 27.0% overall) of confirmed allergies than within the original study (9.3%).^6^

Several studies have recently questioned the NPV of PEN‐FAST and reported misclassification of severe allergic reactions.^11^, ^12^, ^13^ In these studies, most patients misclassified by PEN‐FAST were identified as such due to their positive findings in skin tests, and only a small fraction of patients had a positive DPT. While showing high NPV, there is conflicting data on the positive predictive capacity of penicillin skin testing, especially in study populations with low prevalence.^19^, ^20^, ^21^, ^22^ Intradermal tests are more sensitive than skin prick tests, emphasizing their importance in high‐risk allergies, but they may also show more false positive results.^16^ Our study included only skin prick tests and patch tests and excluded intradermal tests. The latter may have influenced the discrepancies observed regarding the NPV in previous studies. Since all patients misclassified by PEN‐FAST in our retrospective cohort were defined as allergic based on positive patch tests, we further limited skin testing to prick tests only in our prospective cohort to eliminate the potential impact of false‐positive patch tests. This further increased the NPV of PEN‐FAST from 94.5% in the retrospective cohort (n = 55) to 97.5% in the prospective group (n = 40). The effect of false‐positive skin tests on the NPV of PEN‐FAST remains elusive, and only studies with direct DPT will accurately determine the true NPV of PEN‐FAST.

Although a slightly lower NPV than 95% may be acceptable, a recent large‐cohort study reported that PEN‐FAST misclassified high‐risk individuals.^11^ This finding led to questioning the safety of PEN‐FAST and contrasts with a randomized controlled trial in which no severe reactions were observed.^7^ Recent findings emphasize that direct DPT is safe and does not increase the rate of allergic reactions.^15^, ^23^ Several clinical decision rules for penicillin allergies have been reported in recent years, which differ in their predictive capacity, effectiveness, and misclassification of high‐risk subjects.^11^ While other strategies may show higher safety than the PEN‐FAST score, they more frequently lead to unnecessary avoidance.^11^ In the present study, no individuals with any severe allergic reaction (neither in the patient history nor after the drug provocation test) were misclassified by PEN‐FAST, whereas conventional allergy testing failed to identify one patient who developed airway obstruction following DPT. Thus, the selection of the respective clinical tool should not only be guided by the target population but must also consider the performance of the available testing methods, which may also fail to identify high‐risk individuals.

### Limitations

This study has several limitations. Most patients (78.8%) were analyzed in a retrospective, observational design. Limited information on the PEN‐FAST treatment category has been described previously.^11^ In the present study, information on treatment was unavailable in 63.1% of retrospective patients (data not shown). In line with the original study, one point was assigned if information on treatment was unknown or unavailable.^6^ Thus, patients who reported that they had not received any treatment may have been assigned to the PEN‐FAST group of 3–5 due to lack of documentation. This would affect retrospective patients with a PEN‐FAST score of 2 points (n = 38), of which 19 were labeled allergic (data not shown). However, the proportion of patients in our prospective cohort who did not remember if a treatment had been performed was also high (47.5%). Further, no intradermal tests were performed, which may have hindered the potential identification of patients receiving DPT and exhibiting an allergic reaction.

## CONCLUSIONS

The results of our study advocate for PEN‐FAST as a safe and feasible risk‐stratification method to remove penicillin allergy labels in high‐prevalence populations. Prospective studies with direct provocation tests and larger cohorts are needed to further validate the safety and efficacy of PEN‐FAST unaffected by potential false‐positive skin tests.

## FUNDING

Deniz Göcebe and Alexander H. Enk receive funding from grant SFB/TRR 156 of the DFG (Deutsche Forschungsgemeinschaft). Katharina S. Kommoss is funded by the physician scientist program, medical faculty, University of Heidelberg, Heidelberg, Germany.

## CONFLICT OF INTEREST STATEMENT

None.

## Supporting information



Supplementary information
