# Supplementary material for: Implementing PEN‐FAST for penicillin allergy delabeling in a high‐prevalence population
Source: J Dtsch Dermatol Ges. 2025 Sep 14;24(1):57–63. doi: 10.1111/ddg.15862 (PMC12800881; doi:10.1111/ddg.15862)
Supplement: Supplementary file 1 — Supplementary information [file DDG-24-57-s001.pdf]

eAppendix 1

1.1 Inclusion criteria

1. Adult outpatients or inpatients referred to the Allergology Department of the University Hospital of Heidelberg, Germany
2. A fully completed allergy assessment (included patients must have either tested positive by skin testing and/or allergen-specific IgE or received a drug provocation test with the culprit penicillin)
3. Capacity to consent
4. Only in the prospective cohort: PEN-FAST score of < 3 points

1.2 Exclusion criteria

1. Age <18 years
2. Concurrent immunosuppressive therapy with 20 mg of prednisolone per day or steroid equivalent
3. Concurrent antihistamine therapy
4. Pregnancy
5. Skin disease in the test area of skin tests
6. Significantly impaired general condition
7. Unstable or therapeutically inadequately controlled bronchial asthma
8. History of stem cell transplantation
9. History of acute interstitial nephritis
10. Chronic urticaria
11. Mastocytosis

1.3 Determination of the PEN-FAST score

PEN-FAST scores (0-5 points) were determined according to the original study by Trubiano et al.[1] Two points were assigned if the (supposed) allergic reaction occurred in the last five years. Two points were allocated for anaphylaxis, angioedema, or severe cutaneous adverse

reactions (SCAR). One point was assigned if systemic therapy or hospitalization was required to treat the (supposed) allergic reaction.

1.4 Anaphylaxis

Anaphylaxis was defined according to the original study[1] and required a cutaneous manifestation accompanied by at least one of the following: respiratory, cardiovascular, or gastrointestinal symptoms. Anaphylaxis was also determined if there was an acute onset of hypotension, bronchospasm, or airway obstruction without cutaneous manifestation.[1]

1.5 Severe cutaneous adverse reactions

Following the original study,[1] severe cutaneous adverse reactions (SCAR) included SJS/TEN, AGEP, and DRESS. As suggested by Trubiano et al.,[1] patients with potential severe cutaneous adverse reactions also received two points if cutaneous manifestations were accompanied by mucosal ulceration.

1.6 Skin tests

Skin tests were performed following EAACI recommendations[2] and German guidelines.[3]
Skin prick test concentrations were 10,000 IU/ml for penicillin G and 20 mg/ml for amoxicillin, ampicillin, piperacillin, and flucloxacillin. Sodium chloride 0.9% was used as a negative control. Histamine dihydrochloride containing 1mg/ml histamine (Allergopharma, Germany) was used as a positive control. Skin prick tests were read out after 20 minutes. A wheal ≥ 2 mm wheal compared to the negative control was considered positive.
Patch tests were performed using Finn Chambers (SmartPractice, Germany), filled with 17 µl of the culprit penicillin. Patch tests were read out after 48 and 72 hours. Sodium chloride 0.9% was used as a negative control. Sodium lauryl sulfate 0.25% was used as a positive control. Patch tests were considered positive if both erythema and infiltration were present.

1.7 Total serum IgE and Allergen-specific IgE

Allergen-specific IgE and total serum IgE were measured using fluorescent-enzyme-immunoassays (ImmunoCAP, Thermo-Fisher) for penicillin G, penicillin V, amoxicillin, and ampicillin. For each penicillin, a concentration of ≥0.35 kU/l was considered positive.

1.8 Drug provocation test

Drug provocation tests were performed using the culprit penicillin. Patients who reported an allergy to penicillin G received an oral challenge with penicillin VK. A 2-step challenge (50%-50%) was performed with an interval of 30 minutes. The therapeutical dose was 1.2 million units of penicillin VK, 500 mg of amoxicillin, and 250 mg of flucloxacillin, respectively. Patients who reported an allergy to ampicillin or piperacillin received intravenous challenge with a full therapeutical dose of ampicillin/sulbactam 2g/1g and piperacillin/tazobactam 4g/0,5g, respectively. The decision on whether the testing in the retrospective group was conducted on an outpatient or inpatient basis was made after individual consultation with the patient. All prospective patients were tested on an inpatient basis.

1.9 Allergy questionnaire in the prospective cohort

1. Which year did the symptoms first occur, and when did they last occur?
2. What symptoms occurred?

   Skin rash without blistering (yes/no)

   Urticaria (yes/no)

   Cardiovascular symptoms (yes/no)

   Loss of consciousness (yes/no)

   Edema (swelling) (yes/no)

   Gastrointestinal symptoms (yes/no)

   Shortness of breath (yes/no)
3. Have similar symptoms occurred without taking any medication?
4. How long was the period between taking the medication and the onset of symptoms?
5. Has penicillin been used again since the first reaction? If yes, did you tolerate it?

6. Do you have atopic dermatitis?
7. Do you have any other known allergies?
8. Do/did your relatives have similar allergies?
9. Have you ever been prescribed a different antibiotic than the one originally planned because of your penicillin allergy?
10. Have you already undergone an allergy test? If yes, when and which one?
11. Do you smoke?

1.10 Data Collection in the Retrospective Cohort

The patients were identified in the hospital's internal patient management system by searching the relevant worklists of the Allergy Department for the respective ICD codes for penicillin allergies. The complete records for each patient were then reviewed individually.

eFigure 1. Outcomes of Evaluation of penicillin allergy assessment in the **(A)** retrospective and **(B)** prospective cohorts.

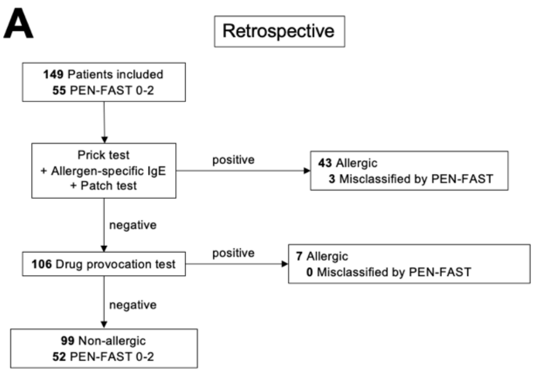

eTable 1. Characteristics of Misclassified Patients.

**Patient**	1	2	3	4	5	6	7	8	9
**Misclassification by**	Conventional testing	Conventional testing	Conventional testing	Conventional testing	Conventional testing	PEN-FAST + Conventional testing	PEN-FAST	PEN-FAST	PEN-FAST
**Sex**	Female	Female	Female	Female	Female	Female	Female	Female	Female
**Age**	44	36	79	33	34	76	45	20	32
**Reported allergy label**	Amoxicillin	Amoxicillin	Penicillin VK/G	Penicillin VK/G	Penicillin VK/G	Penicillin VK/G	Amoxicillin	Amoxicillin	Amoxicillin
**Symptom**	Airway obstruction, tachycardia	Urticaria/rash	Urticaria/rash	Urticaria/rash	Urticaria/rash	Urticaria/rash	Urticaria/rash	Urticaria/rash	Urticaria/rash
**Onset latency**	Few hours	Unclear/unkown	Unclear/unkown	2-3 days	3 days	Unclear/unkown	Unclear/unkown	1 day	Few hours
**Allergy type**	Immediate	Unclear/unkown	Unclear/unkown	Delayed	Delayed	Unclear/unkown	Unclear/unkown	Delayed	Immediate
**PEN-FAST score**	3	3	3	3	3	1	1	2	1
**Five years or less since reaction**	no	yes	yes	yes	yes	no	no	yes	no
**Anapyhlaxis/Angiodema or SCAR**	yes	no	no	no	no	no	no	no	no
**Treatment required for reaction**	Unclear/unkown	Unclear/unkown	Unclear/unkown	Unclear/unkown	Unclear/unkown	Unclear/unkown	Unclear/unkown	No	Unclear/unkown
**Allergen-specific IgE (kU/l)**	< 0.35	< 0.35	< 0.35	< 0.35	< 0.35	< 0.35	< 0.35	< 0.35	< 0.35
**Total serum IgE**	3.4	61	30.9	119	8.5	54	79	178	11.3
**Specific/total IgE ratio**	NA	NA	NA	NA	NA	NA	NA	NA	NA
**Prick test**	Negative	Negative	Negative	Negative	Negative	Negative	Negative	Negative	Negative
**Patch test**	Negative	Not performed	Negative	Negative	Negative	Not performed	Positive	Positive	Positive
**Drug provocation test result**	Positive (Airway obstruction)	Positive (Urticaria/rash)	Positive (Urticaria/rash)	Positive (Urticaria/rash)	Positive (Urticaria/rash)	Positive (Urticaria/rash)	Not performed	Not performed	Not performed

NA, not applicable; SCAR, severe cutaneous adverse reaction.

eTable 2. Outcomes of Total Serum IgE, Tryptase, and SX1 Inhalant Allergens.
Mean (±SD).

	Total IgE (kU/l)	p-value*	Tryptase (µg/l)	p-value*	SX1 inhalent allergens (kU/l)	p-value*
Total (n=189)	140.5 (±426.3)	NA	5.4 (±3.1)	NA	4.9 (±13.4)	NA
Allergic (n=51)	293.1 (±949.2)	**0.0328**	5.3 (±2.9)	0.8588	8.1 (±20.0)	0.8588
Non-allergic (n=138)	76.9 (±106.8)		5.5 (±3.2)		4.0 (±10.1)	
PEN-FAST 0-2 (n=95)	85.3 (±107.8)	0.9402	5.8 (±3.5)	0.8013	3.7 (±9.0)	0.8013
PEN-FAST 3-5 (n=94)	168.6 (±661.8)		5.1 (±2.6)		6.3 (±16.5)	

NA, not applicable.
*Kruskal-Wallis; bold values indicate statistical significance (P < .05).

eTable 3. Characteristics of Patients with Elevated Allergen-specific IgE.

**Patient number**	1	2	3	4	5	6	7	8	9
**Sex**	Male	Female	Female	Male	Female	Female	Male	Male	Male
**Age**	76	64	73	18	59	18	18	28	51
**Reported allergy label**	Penicillin VK/G	Penicillin VK/G	Amoxicillin	Penicillin VK/G	Penicillin VK/G	Penicillin VK/G	Penicillin VK/G	Amoxicillin	Amoxicillin
**Symptom**	Urticaria/rash	Urticaria/rash	Angioedema	Urticaria/ rash, hypotension	Airway obstruction, skin erosion	Angioedema, airway obstruction	Urticaria/rash	Urticaria/rash, angioedema	Angioedema, Airway obstruction
**Onset latency**	7 days	Unclear/unkown	Unclear/unkown	Minutes	Minutes	Minutes	10 days	8 days	Minutes
**Allergy type**	Delayed	Unclear/unkown	Immediate	Immediate	Immediate	Immediate	Delayed	Delayed	Immediate
**PEN-FAST score**	3	3	5	5	3	5	3	5	5
**Five years or less since reaction**	yes	yes	yes	yes	no	yes	yes	yes	yes
**Anapyhlaxis/Angiodema or SCAR**	no	no	yes	yes	yes	yes	no	yes	yes
**Treatment required for reaction**	Unclear/unkown	Oral corticosteroids/ antihistamines	Unclear/unkown	Unclear/ unkown	Unclear/unkown	Oral corticosteroids/ antihistamines	Oral corticosteroids/ antihistamines	Oral corticosteroids/ antihistamines	Oral corticosteroids/ antihistamines
**Allergen-specific IgE (kU/l)**	0.98	0.86	3.65	1.85	0.4	75.3	0.51	2.31	6.68
**Total serum IgE**	1892.0	42.1	4.3	185.0	1383.0	205.0	159.0	8.5	282.0
**Specific/total IgE ratio**	**0.00052**	0.02043	0.85082	0.01000	**0.00029**	0.36732	0.00321	0.27176	0.02369
**Prick test**	Not performed	Negative	Not performed	Not performed	Negative	Not performed	Not performed	Negative	Negative
**Patch test**	Not performed	Not performed	Not performed	Not performed	Negative	Not performed	Negative	Positive	Negative
**Drug provocation test result**	Not performed	Not performed	Not performed	Not performed	Negative	Not performed	Not performed	Not performed	Not performed

SCAR, severe cutaneous adverse reaction.
Bold values indicate specific/total IgE ratio values <0.002 as previously described[4]

eTable 3. (continued)

Patient number	10	11	12	13	14	15	16	17	18
**Sex**	Female	Female	Male	Male	Male	Female	Female	Female	Female
**Age**	49	52	44	54	40	37	31	55	59
**Reported allergy label**	Amoxicillin	Penicillin VK/G	Amoxicillin	Amoxicillin	Amoxicillin	Penicillin VK/G	Amoxicillin	Amoxicillin	Amoxicillin
**Symptom**	Urticaria/rash	Urticaria/rash, hypotension	Urticaria/rash, blistering, hypotension	Urticaria/rash	Urticaria/rash	Angioedema	Urticaria/rash, hypotension	Angioedema	Airway obstruction
**Onset latency**	5 days	Unclear/unkown	Few hours	6 days	9 days	Minutes	Minutes	Minutes	Minutes
**Allergy type**	Delayed	Unclear/unkown	Immediate	Delayed	Delayed	Immediate	Immediate	Immediate	Immediate
**PEN-FAST score**	3	5	5	3	3	3	5	5	5
**Five years or less since reaction**	yes	yes	yes	yes	yes	no	yes	yes	yes
**Anapyhlaxis/Angiodema or SCAR**	no	yes	yes	no	no	yes	yes	yes	yes
**Treatment required for reaction**	Oral corticosteroids/ antihistamines	Unclear/unkown	IV corticosteroids/ antihistamines	Unclear/unkown	Oral corticosteroids/ antihistamines	Unclear/unkown	Oral corticosteroids/ antihistamines	Oral corticosteroids/ antihistamines	Unclear/unkown
**Allergen-specific IgE (kU/l)**	0.35	3.9	0.46	8.78	12.7	0.35	1.92	2.8	0.43
**Total serum IgE**	32.0	1203.0	5000.0	474.0	390.0	9.0	224.0	549.0	152.0
**Specific/total IgE ratio**	0.01094	0.00324	**0.00009**	0.01852	0.03256	0.03889	0.00857	0.00510	0.00283
**Prick test**	Positive	Negative	Negative	Not performed	Positive	Not performed	Not performed	Positive	Not performed
**Patch test**	Not performed	Negative	Negative	Not performed	Negative	Not performed	Not performed	Negative	Not performed
**Drug provocation test result**	Not performed	Not performed	Positive (Urticaria/rash, angioedema))	Not performed	Not performed	Not performed	Not performed	Not performed	Not performed

SCAR, severe cutaneous adverse reaction.
Bold values indicate specific/total IgE ratio values <0.002 as previously described[4]

eReferences

E1. Trubiano JA, Vogrin S, Chua KYL, Bourke J, Yun J, Douglas A, et al. Development and Validation of a Penicillin Allergy Clinical Decision Rule. JAMA Intern Med. 2020;180(5):745-52.
E2. Romano A, Atanaskovic-Markovic M, Barbaud A, Bircher AJ, Brockow K, Caubet JC, et al. Towards a more precise diagnosis of hypersensitivity to beta-lactams - an EAACI position paper. Allergy. 2020;75(6):1300-15.
E3. Wurpts G, Aberer W, Dickel H, Brehler R, Jakob T, Kreft B, et al. S2k-Leitlinie: Diagnostik bei Verdacht auf eine Betalaktamantibiotika-Überempfindlichkeit: Leitlinie der Deutschen Gesellschaft für Allergologie und klinische Immunologie (DGAKI) in Zusammenarbeit mit dem Ärzteverband Deutscher Allergologen (AeDA), der Gesellschaft für Pädiatrische Allergologie und Umweltmedizin (GPA), der Deutschen Kontaktallergiegruppe (DKG), der Österreichischen Gesellschaft für Allergologie und Immunologie (ÖGAI) und der Paul-Ehrlich-Gesellschaft für Chemotherapie (PEG). Allergo Journal. 2019;28:19-51.
E4. Vultaggio A, Virgili G, Gaeta F, Romano A, Maggi E, Matucci A. High serum β-lactams specific/total IgE ratio is associated with immediate reactions to β-lactams antibiotics. PLoS One. 2015;10(4):e0121857.
